# Biocompatible Silk/Polymer Energy Harvesters Using Stretched Poly (vinylidene fluoride-*co*-hexafluoropropylene) (PVDF-HFP) Nanofibers

**DOI:** 10.3390/polym9100479

**Published:** 2017-09-30

**Authors:** Raghid Najjar, Yi Luo, Dave Jao, David Brennan, Ye Xue, Vince Beachley, Xiao Hu, Wei Xue

**Affiliations:** 1 Mechanical Engineering, Rowan University, Glassboro, NJ 08028, USA; najjarr2@students.rowan.edu; 2 Communication Engineering, Hangzhou Dianzi University, Hangzhou 310018, China; luoyi@hdu.edu.cn; 3 Biomedical Engineering, Rowan University, Glassboro, NJ 08028, USA; jaod07@students.rowan.edu (D.J.); brenna92@students.rowan.edu (D.B.); xuey5@rowan.edu (Y.X.); beachley@rowan.edu (V.B.); hu@rowan.edu (X.H.); 4 Physics and Astronomy, Rowan University, Glassboro, NJ 08028, USA

**Keywords:** energy harvester, polyvinylidene fluoride (PVDF), silk, piezoelectricity, electrospinning, stretching

## Abstract

Energy harvested from human body movement can produce continuous, stable energy to portable electronics and implanted medical devices. The energy harvesters need to be light, small, inexpensive, and highly portable. Here we report a novel biocompatible device made of poly (vinylidene fluoride-*co*-hexafluoropropylene) (PVDF-HFP) nanofibers on flexible substrates. The nanofibers are prepared with electrospinning followed by a stretching process. This results in aligned nanofibers with diameter control. The assembled device demonstrates high mechanical-to-electrical conversion performance, with stretched PVDF-HFP nanofibers outperforming regular electrospun samples by more than 10 times. Fourier transform infrared spectroscopy (FTIR) reveals that the stretched nanofibers have a higher β phase content, which is the critical polymorph that enables piezoelectricity in polyvinylidene fluoride (PVDF). Polydimethylsiloxane (PDMS) is initially selected as the substrate material for its low cost, high flexibility, and rapid prototyping capability. Bombyx Mori silkworm silk fibroin (SF) and its composites are investigated as promising alternatives due to their high strength, toughness, and biocompatibility. A composite of silk with 20% glycerol demonstrates higher strength and larger ultimate strain than PDMS. With the integration of stretched electrospun PVDF-HFP nanofibers and flexible substrates, this pilot study shows a new pathway for the fabrication of biocompatible, skin-mountable energy devices.

## 1. Introduction

The demand for wearable electronics and sensors has been continuously increasing in the past decade. This trend has resulted in many innovative devices such as wrist bands, smart watches, and embedded sensors in sportswear. These devices generally rely on batteries to provide the energy needed for them to function. However, the battery technology has not been advancing at the same pace as consumer electronics [1,2,3]. Energy harvesting, the act of acquiring energy from the environment and converting it to a useful form [4], is considered a great alternative to batteries or at least a secondary energy source to extend the battery life. Before the energy harvesters can be used in commercial devices, they need to fulfill some essential requirements. The devices have to be small, light, inexpensive, portable, and flexible. These ergonomic requirements increase the chance of the devices’ marketability. Furthermore, these energy harvesters have to be highly biocompatible for critical applications in the medical field, such as for pacemakers and automatic implantable cardioverter defibrillators (AICD).

Piezoelectric materials contain crystals that become electrically polarized under applied stresses [5]. This polarization creates a voltage, which can be harvested and used, or stored in a battery, in the material. Commonly used piezoelectric materials for energy harvesting applications include lead zirconate titanate (PZT), BaTiO_3_, ZnO, and polyvinylidene fluoride (PVDF) [6,7]. PZT has a very high electro-mechanical coupling factor of 0.71 [8]. However, the lead content in PZT reduces its biocompatibility [9,10]. BaTiO_3_ and ZnO have high electro-mechanical coupling factors of 0.48 [8,11]. They are promising lead-free piezoelectric materials [9]. These three inorganic materials perform well as energy generators for some applications, but their biocompatibility is relatively limited for wearable electronics. Alternatively, PVDF and its copolymers such as poly (vinylidene fluoride-*co*-hexafluoropropylene) (PVDF-HFP) [12,13,14] and poly (vinylidene fluoride-trifluoroethylene) (PVDF-TrFE) [15,16,17], prepared by solvent casting or electrospinning, offer reasonable electro-mechanical coupling factors between 0.20 and 0.39 [18]. More importantly, PVDF has shown to be a safe biomaterial suitable for medical applications [19]. Most PVDF-based devices are light and flexible. All these qualities grant marketability potential for PVDF energy harvesters.

PVDF energy harvesting is a well-researched subject. Numerous studies that examine the piezoelectric performance of PVDF nanofibers [3,20,21] and further enhance the efficiency of the resulted devices [22] have been reported. The light weight, high flexibility, and long-term stability of PVDF are desired properties for wearable and implantable electronics. Therefore, there has been considerable research on the mechanisms of PVDF harvesting vibration and motion energy. Early studies showed that PVDF coupled with PZT could be used in the insoles of shoes as energy harvesters [23]. More recent applications used only PVDF in the insole of shoes to power pedometers [24]. Another application placed PVDF in the strap of a backpack to harvest the energy generated by its movement [25].

Various types of synthetic polymers such as polydimethylsiloxane (PDMS), poly (methyl methacrylate) (PMMA), and polyethylene terephthalate (PET) have been widely used as the substrate materials for wearable electronics. They provide advantages such as higher flexibility, lower cost, easier processing, and enhanced biocompatibility when compared with traditional rigid substrates such as silicon or glass. Natural proteins such as silk fibroins (SF) can provide even better biocompatibility and more favorable sustainability than synthetic polymers. SF is generally derived from large scale cultivation of silkworms through sericulture. The domesticated mulberry Bombyx Mori (B. Mori) silkworms are the main producers of silk [26]. Mainly cultivated in plantations for high-end textile fibers, the application potential of SF is multidimensional, such as in biotechnology, materials science, medical research, and pharmaceuticals [27]. The versatile processability of SF into different forms such as gels, films, foams, membranes, scaffolds, and nanofibers makes it appealing in a variety of applications that require mechanically superior, biocompatible, biodegradable, and functionalizable biomaterial. With excellent film-forming capabilities, SF could be used in electronic and photonic applications [28]. The multiple functionalities of SF offer a range of modifications as well as a wide spectrum of derivatives for biomedical applications. With favorable interactions in biological systems and minimal immunological responses, SF materials have demonstrated good biocompatibility with various cell types by supporting and promoting adhesion, proliferation, growth, and differentiation of cells leading to tissue regeneration [27]. The advantage of SF being oxygen permeable in the wet state, similar to that of human skin, makes it useful in the development of matrices/morphologies for the delivery of bioactive molecules, growth factors, signaling cues, drug release profiles, and wound dressing [28,29]. Because of all these highly desired properties, SF material is considered as one of the ideal candidates as a substrate for flexible electronics or sensor systems.

Here we report the design, fabrication, and measurement of a novel wearable energy harvester. The device integrates PVDF-HFP nanofibers and flexible substrates for enhanced biocompatibility and durability. Two types of PVDF-HFP nanofibers (with thicker fibers at the microscale) are fabricated: one with a traditional electrospinning process while the other goes through an additional stretching step before the nanofibers fully solidify. The resulting random and stretched nanofibers are characterized for their physical and piezoelectric properties. Electromechanical testing shows that under the same testing condition, the stretched and aligned PVDF-HFP nanofibers outperform the random nanofibers by more than 10 times in terms of energy conversion. Two different materials, PDMS as a synthetic polymer and SF as a natural protein, are investigated as the substrate materials. PDMS is used due to its well-known properties, low material and fabrication costs, high flexibility, and rapid prototyping capability. SF is used for its better biocompatibility and enhanced strength. However, pure SF films tend to be stiff and brittle in the dry state over time, exhibiting impressive tensile strength but poor elongation (or ultimate strain) [30]. A well-documented plasticizer, glycerol, is therefore added to make the SF film more flexible. The mechanical characterization shows that the SF-glycerol composite demonstrates higher strength and larger ultimate strain than the PDMS film. As a proof-of-concept device, our flexible PVDF-HFP energy harvester has demonstrated satisfactory electromechanical performance. It has great potential to be used in a broad range of applications, especially in skin-mountable medical devices or wearable health monitoring sensor systems. We believe that this project can eventually lead to a new growth area of the wearable electronics market. The design, fabrication, and testing of the energy harvester are described and discussed in this paper. The aspects of the materials, including the preparation and characterization of various materials, as well as their impact on the overall device performance, are also discussed.

## 2. Materials and Methods

### 2.1. Polymer Materials

#### 2.1.1. Preparation of PVDF-HFP Solvents and PDMS

A PVDF-HFP copolymer (molecular weight: 400,000, pellets), purchased from Sigma-Aldrich (St. Louis, MO, USA), was used as the piezoelectric polymer. The organic solvent *N*,*N*-Dimethylformamide (DMF) was also purchased from Sigma-Aldrich. The PVDF-HFP solution was prepared by mixing 28 g of PVDF-HFP pellets in 10 mL of DMF. The mixture was placed on a shaker for 24 h for the PVDF-HFP pellets to fully dissolve, resulting in a highly viscous, homogenous solution. The material preparation steps were performed at room temperature. Afterwards, the solution was drawn into a 10 mL syringe which was placed in a syringe pump for electrospinning.

The elastomer PDMS was purchased from Dow Corning (Midland, MI, USA). It came as a two-part kit with a base polymer and a curing agent. The two components were mixed together with a weight ratio of 10:1. The mixed PDMS was placed in a desiccator for 3 h to remove air bubbles. Afterwards, it was casted onto a petri dish and cured at 65 °C for 8 h on a hot plate. The cured PDMS was peeled off from the petri dish and then cut into small pieces.

#### 2.1.2. Electrospinning and Stretching of PVDF-HFP Nanofibers

**Figure 1:**
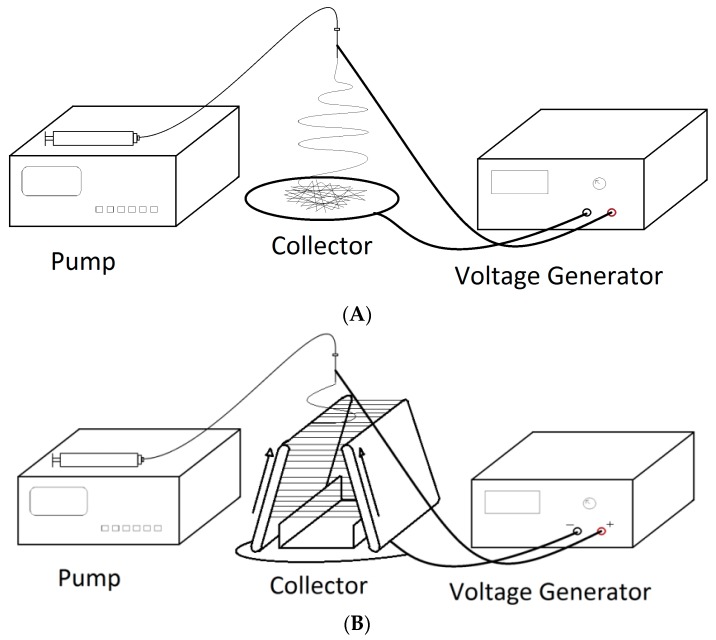
Schematics of (**A**) the electrospinning system to produce random nanofibers and (**B**) the electrospinning system to produce aligned and stretched nanofibers.

The PVDF-HFP nanofibers were obtained through an electrospinning process. The electrospinning system was built in house, based upon a previously demonstrated system [31,32,33]. Figure 1 shows the schematics of the two electrospinning systems used in this project. The PVDF-HFP solution was pumped at a rate of 8 mL/h through a 21-gauge blunt tip needle. A power supply ES40P-10W (Gamma High Voltage Research Inc., Ormond Beach, FL, USA) was used to apply a high voltage of 12,000 V to the needle. The needle was placed approximately 12 cm above an electrically grounded metal collector. The collector, coated with a layer of aluminum foil, was used to collect random PVDF-HFP nanofibers extruded from the needle, as shown in Figure 1A. For stretched nanofibers, the collector was in the form of parallel plates, as demonstrated in previous publications by Beachley and Wen [31,33]. The stretching was achieved through a parallel automated track collector [34]. The system has two conveyor belts and a stationary collector, as shown in Figure 1B. The nanofibers are deposited in parallel between the two belts. Before the solvent fully evaporates and the nanofibers fully solidify, the belts move the fibers down and stretch them along the path. The gap widths between the belts at the top and the bottom are adjustable, and, therefore, the final length of the nanofibers is also controllable. The collected nanofibers were stretched to twice of their original length (from 3 cm to 6 cm) while still in a semi-solid state. The stretched nanofibers were continuously deposited on a collector device between the belts. After the DMF evaporated and the nanofibers solidified, the samples were collected directly on the substrate, PDMS or SF-glycerol composite, for device assembly. 

#### 2.1.3. Scanning Electron Microscopy (SEM) Inspection and Quantitative Analysis of Nanofibers

The electrospun nanofibers were inspected with an SEM, Phenom Pure (Phenom-World, Eindhoven, The Netherlands). The obtained SEM images of the nanofibers were analyzed using image analysis software ImageJ. The diameter distribution and alignment information of the fibers were obtained. The diameter measurements were used to ensure that the electrospun nanofibers had consistent diameters and were within the nanometer range.

### 2.2. Silk Materials

#### 2.2.1. Preparation of Silk and Silk-Glycerol Solutions

Silk cocoons came from Bombyx Mori silkworm (Lakewood, CO, USA) were boiled for 25 min in an aqueous solution of 0.02 M NaHCO_3_ and rinsed thoroughly with Milli-Q water to remove the glue-like sericin proteins. The extracted silk proteins were dried and dissolved into a mixture solution of formic acid and calcium chloride (4 wt % of calcium chloride) for 15 min. After centrifugation and filtration to remove insoluble residues, a 6 wt % SF solution was obtained. To obtain the SF-glycerol solution, glycerol powders were directly added into the SF solution at the mass ratio of 1:4 (glycerol/silk). The final solutions (either SF or SF-glycerol composite, 15–20 mL) were casted onto pre-cured PDMS substrates to form regenerated films. After being rinsed in distilled water several times, the dried biocompatible SF or SF-glycerol films were obtained.

#### 2.2.2. Fourier Transform Infrared Spectroscopy (FTIR) Analysis

FTIR analysis was performed using a Bruker FTIR Spectrometer Tensor 27 (Bruker Co. Ltd., Billerica, MA, USA), which was equipped with a deuterated triglycine sulfate detector and a multiple-reflection, horizontal MIRacle attenuated total reflection (ATR) attachment (using a Ge crystal). For each measurement, 128 scans were co-added with a resolution of 4 cm^−1^, with the wavenumber ranging from 400 to 4000 cm^−1^. Fourier self-deconvolution (FSD) of the IR spectra covering the amide I region (1595–1705 cm^−1^) was performed by Opus 5.0 software. Deconvolution was performed using Lorentzian line shape with a half-bandwidth of 25 cm^−1^ and a noise reduction factor of 0.3. FSD spectra were then curve-fitted to measure the relative areas in the amide I region.

#### 2.2.3. Differential Scanning Calorimetry (DSC) Analysis

The dried samples were encapsulated in aluminum pans and heated in a TA Instrument Q100 DSC (New Castle, DE, USA), with a purged dry nitrogen gas flow of 50 mL/min and equipped with a refrigerated cooling system. Each sample weighed approximately 5 mg. The instrument was calibrated with indium for heat flow and temperature. Aluminum and sapphire reference standards were used for the calibration of the heat capacity. Standard mode DSC measurements were performed at a heating rate of 2 °C/min. Temperature modulated differential scanning calorimetry (TMDSC) measurements were performed at a heating rate of 2 °C/min with a modulation period of 60 s and a temperature amplitude of 0.318 °C. Through TMDSC, the “reversing heat capacity”, which represents the reversible heat effect of samples within the temperature range of the modulation, can be measured and calculated.

#### 2.2.4. SEM Imaging

The PDMS, SF, and SF-glycerol composite samples were examined using the Phenome Pure SEM (Phenom-World, Eindhoven, The Netherlands). The obtained SEM images show the morphologies of these samples.

### 2.3. Mechanical Property Characterization

The mechanical properties of the PDMS, pure SF, and SF-glycerol composite films were investigated using the stress-strain curve method, obtained by a tensile tester Shimpo FGS-200PV (ELECTROMATIC Equipment Co., Cedarhurst, NY, USA). All testing samples were cut into small pieces to fit into the tensile machine. Each sample was stretched under an increasing tensile force starting at 0 N with 0.1 N increments (1 N increments for the pure SF sample due to its high strength). The sample was continuously stretched until it broke into two pieces. The real-time force and elongation data of the sample were recorded. Afterwards, the stress and strain values for each sample were calculated using the applied tensile force, length of the sample, and the cross sectional area of the sample.

### 2.4. Energy Harvester Design and Assembly

**Figure 2:**
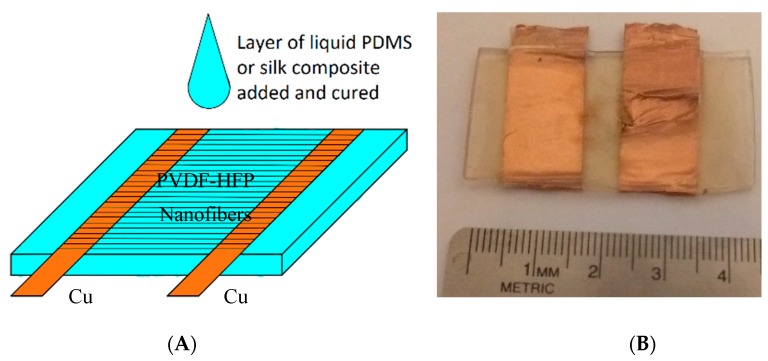
(**A**) Schematic of the device design and fabrication. (**B**) A sample PDMS/PVDF-HFP/PDMS device. PVDF-HFP = poly (vinylidene fluoride-co-hexafluoropropylene); PDMS = polydimethylsiloxane.

The initial design of the energy harvester used PDMS as the substrate. The assembly of the device involved both pre-cured and un-cured, liquid PDMS, as illustrated in Figure 2. The pre-cured PDMS was cut into pieces with dimensions of 45 mm (length) × 25 mm (width) × 1 mm (thickness). Two copper strips were placed horizontally on opposite sides of the PDMS substrate as electrical leads of the final device. While the PVDF-HFP nanofibers were still in the collector plates, the top side of the PDMS piece and the copper strips were brought into a direct contact with the nanofibers. A razor blade was used to cut around the PDMS substrate to free the PVDF-HFP/copper/PDMS assembly. Afterwards, the device was placed in a tight container and un-cured; liquid PDMS was poured on it to cover the entire top surface. The liquid PDMS bound seamlessly with the bottom PDMS substrate as well as filled in all the cavities of the PVDF-HFP nanofiber network. This device was heated on a hot plate, set at 65 °C for 8 h, for the liquid PDMS to cure. The silk-based device was fabricated following a similar procedure, combining a pre-cured substrate and liquid silk on top of the nanofibers. An assembled PDMS/PVDF-HFP/PDMS device is shown in Figure 2. The gap between the two copper strips is approximately 5 mm. The overall dimensions of the device as well as the copper strips can be further miniaturized for smaller systems.

### 2.5. Mechanical-Electrical Experimental System Setup

**Figure 3:**
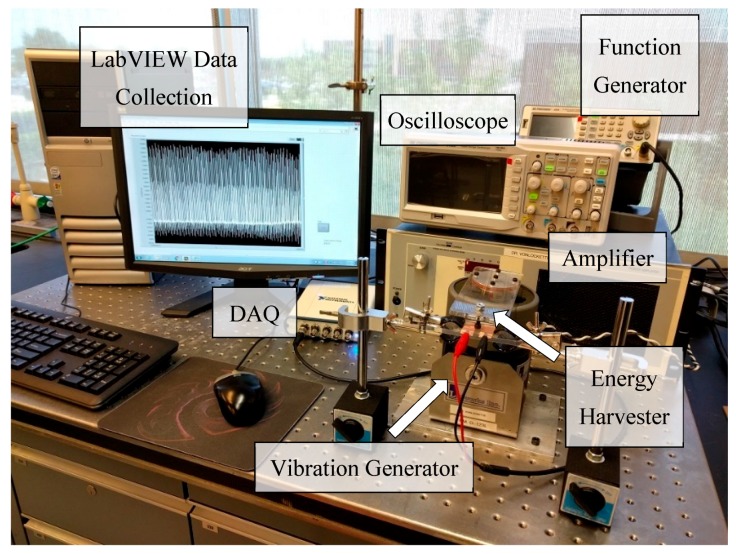
The mechanical-electrical measurement system for the energy harvester. DAQ = data acquisition system.

A mechanical-electrical measurement system, as shown in Figure 3, was used to investigate the energy harvesting performance of the device. A controlled vibration was introduced by a vibration system LW126 (Labworks Inc., Costa Mesa, CA, USA). A function generator BK Precision 4054 (B&K Precision Co., Yorba Linda, CA, USA) was used to supply a sinusoidal wave with an amplitude of 250 mV to a linear power amplifier PA-141 (Labworks Inc., Costa Mesa, CA, USA) that amplified the signal and then sent it to an electrodynamic magnet shaker ET-126B (Labworks Inc.). A thick, rectangular acrylic plate was bolted on the shaker’s surface with one end cantilevered out as the vibration generator. The PVDF-HFP energy harvester was placed on a fixed hard surface directly underneath the cantilever. During testing, the vibration generator repeatedly tapped on the front surface of the PVDF-HFP device. The force from the shaker was applied orthogonally to the nanofiber plane. The generated electrical signals were inspected with an oscilloscope BK Precision 2190D (B&K Precision Co.) as well as collected by a data acquisition system (DAQ) NI USB-4431 (National Instruments Co., Austin, TX, USA). A visual measurement system, developed in LabVIEW, was used to acquire and analyze the output electrical signals from the device and display them on a computer monitor. The recorded data indicated the electrical performance of the device. In particular, the peak-to-peak voltage quantified the mechanical-to-electrical conversion performance of the energy harvester.

## 3. Results and Discussion

### 3.1. Material Characterization

#### 3.1.1. SEM Imaging

**Figure 4:**
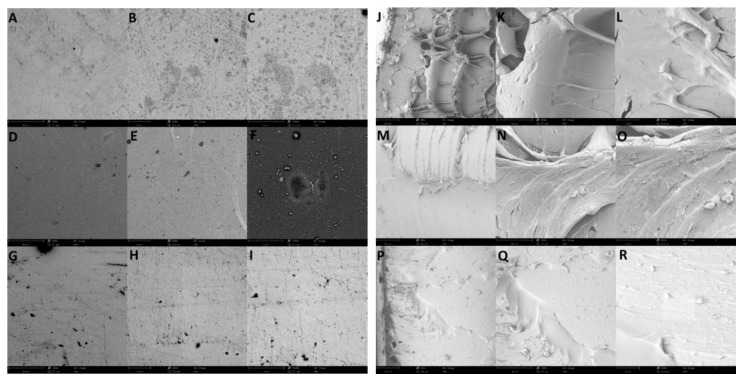
(**A**–**I**) SEM images showing the surface morphology of (**A**–**C**) pure silk fibroin, (**D**–**F**) silk fibroin with 20% glycerol content, and (**G**–**I**) pristine PDMS films at 1000× (scale bar of 80 µm), 5000× (scale bar of 10 µm), and 10,000× (scale bar of 8 µm), respectively. (**J**–**R**) SEM images showing the cross-sections of (**J**–**L**) pure silk fibroin, (**M**–**O**) silk fibroin with 20% glycerol content, and (**P**–**R**) pristine PDMS at 1000× (scale bar of 80 µm), 5000× (scale bar of 10 µm), and 10,000× (scale bar of 8 µm), respectively.

The surfaces and cross-sections of the regenerated SF, SF-glycerol composite, and PDMS films were observed with an SEM. Figure 4A–I show the top-view SEM images of the pure SF, SF with 20% glycerol content, and PDMS samples. These images illustrate the surface microstructures and morphologies of the three materials. In particular, Figure 4A–C illustrate the pure SF film with uniform NaCl crystals from the acid digestion process. The SEM images in Figure 4D–F show evenly distributed NaCl crystals of uniform sizes in the SF-glycerol composite. No significant changes are found along the depth of the film, indicating homogeneous gel formation. The SEM images in Figure 4G–I show the pristine PDMS cross-linked microstructure. The PDMS presents no significant change in morphology. The cross-sectional images of the three samples, pure SF, SF-glycerol composite, and pristine PDMS, are shown in Figure 4J–R. All three materials show continuous and homogeneous structures without voids. The rough cross-sections indicate the tenacity fracture of the films that are related to the strong mechanical properties.

The 20% glycerol in SF solution was selected to maximize the flexibility without over saturating the SF film, which could cause phase separation at the macroscale. Glycerol concentrations lower than 5% did not significantly change the silk film’s mechanical properties. When the glycerol content was increased to 10%, the silk film was flexible. However, it would become brittle after soaking in water, behaving similarly to films that contained no glycerol. A glycerol concentration greater than 20% could swell the SF film. This could cause the glycerol to phase separate to the surface of the film and eventually be lost in water when soaked. Therefore, the 20% glycerol was determined to be an optimal concentration in terms of inducing significant increase to silk film softness, without unnecessary film disassembly in water. As a result of new intermolecular bond formation and augmentation of the β-sheet structure, the new SF-glycerol composite films exhibit a significant increase in flexibility with a small decrease in thermal stability due to the glycerol component (Supplemental material, Figures S1 and S2).

#### 3.1.2. FTIR Analysis

**Figure 5:**
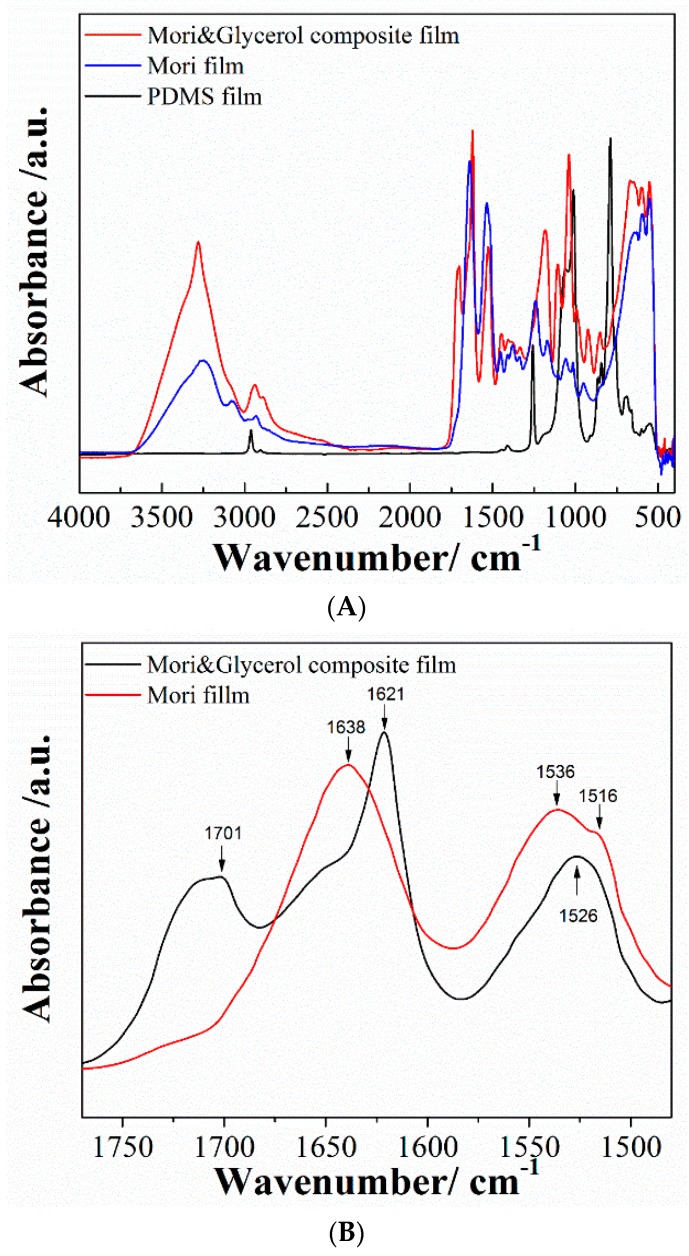
(**A**) FTIR spectra of the PDMS, pure silk fibroin (SF), and SF-glycerol composite films in the range of 4000–450 cm^−1^. (**B**) FTIR spectra of the pure SF and SF-glycerol composite in the range of 1800–1450 cm^−1^.

FTIR spectra of the pristine PDMS, pure SF, and SF-glycerol composite films in the range of 4000–450 cm^−1^ are shown in Figure 5A. The spectra for the PDMS film exhibits its typical characteristic IR bands. The PDMS exhibits absorption at 788–796 cm^−1^ (–CH_3_ rocking and Si–C stretching in Si–CH_3_), 1020–1100 cm^−1^ (Si–O–Si stretching), 1260–1258 cm^−1^ (CH_3_ deformation in Si–CH_3_), and 2950–2960 cm^−1^ (asymmetric CH_3_ stretching in Si–CH_3_).

The SF protein exists mainly in three conformations, namely, random coil, α-helix, and β-sheet conformations. The strong absorptions at 1638 cm^−1^ for amide I (C=O stretching), 1530 cm^−1^ for amide II (N–H deformation), and 1230 cm^−1^ for amide III (C–N stretching, C=O bending vibration) are observed from the pure SF film group, which indicates that it only contains random coil and α-helical conformations. The spectra of the SF-glycerol composite showed shifts in the absorption band of amide I from 1638 cm^−1^ (pure silk) to a lower wavenumber (1621 cm^−1^), reflecting that the silk molecules formed insoluble β-sheet structure in the SF-glycerol composite after interlinking with the glycerol molecules (Figure 5B). The amide II and amide III bands are also shifted to 1526 cm^−1^ and 1170 cm^−1^, respectively, illustrating augmentation of the β-sheet structure. The absorption band appearing at 1230 cm^−1^ (amide III) represents a mixed vibration of CO–N and N–H. Moreover, a new absorption band appears at 1701 cm^−1^ for carboxylic acids (C=O) in the SF-glycerol composite film, which further verifies the new intermolecular hydrogen bonds among SF molecules.

#### 3.1.3. Mechanical Property Characterization

**Figure 6:**
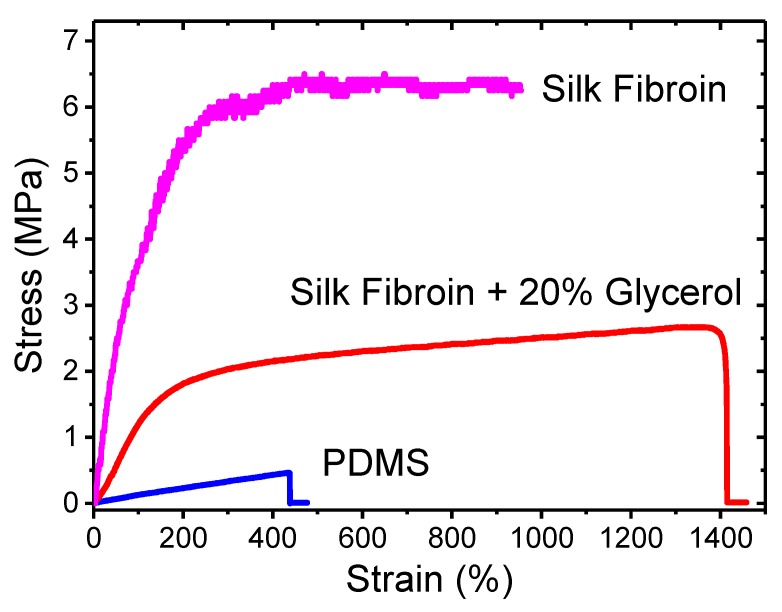
Stress-strain curves of the PDMS, pure SF, and SF-glycerol composite films.

The stress-strain curves of the three materials are plotted into one graph for direct comparison, as shown in Figure 6. There are significant differences in terms of their mechanical behaviors. A table summarizing their key mechanical parameters can be found in Table S1 in the Supplemental material. The PDMS sample is mostly elastic without showing much plasticity. Its stress-strain curve (in blue, the lowest curve of the three) is highly linear until the final fracture point. The sample can be stretched up to 437% of its original length. The key mechanical parameters of the PDMS are obtained as 106.2 kPa (Young’s modulus), 464.0 kPa (yield strength and ultimate strength), and 4.3675 (yield strain and ultimate strain).

In contrast, the stress-strain curve of the pure SF sample (in purple, the highest of the three) shows a large deviation from that of the PDMS. It shows clear elastic and plastic regions with drastically enhanced strength values. The Young’s modulus of the pure SF is calculated as 4.7892 MPa. The yield strain and strength are estimated as 0.4350 and 2.0833 MPa, respectively. These parameters indicate that the pure SF film is stiffer than PDMS and has a lower yield point. The ultimate strength and strain, however, are at higher values of 6.25 MPa and 9.5450, respectively. Based on our experience handling the silk materials, completely dried SF samples are usually brittle. Their integration with other functional polymers in flexible devices can be challenging.

The SF-glycerol composite sample demonstrates a balanced mechanical behavior in terms of flexibility and strength. The stress-strain curve (in red, the middle curve) of the SF-glycerol composite is between the curves of PDMS and pure SF. The estimated yield strength of 1.3684 MPa and ultimate strength of 2.6711 MPa demonstrate that the SF-glycerol composite is a stronger material than PDMS. Its ultimate strain of 13.6375 is also significantly higher than that of the PDMS. These parameters prove that the SF-glycerol composite can survive larger forces and greater elongation. One drawback is that the SF-glycerol composite is stiffer than the PDMS film, with a Young’s modulus of 1.1621 MPa. Its elastic range of 1.1775 (yield strain) is also smaller. Nonetheless, the SF-glycerol composite is still a much softer material compared with the pure SF films and its mechanical properties are more suitable for wearable applications.

### 3.2. PVDF-HFP Nanofibers

**Figure 7:**
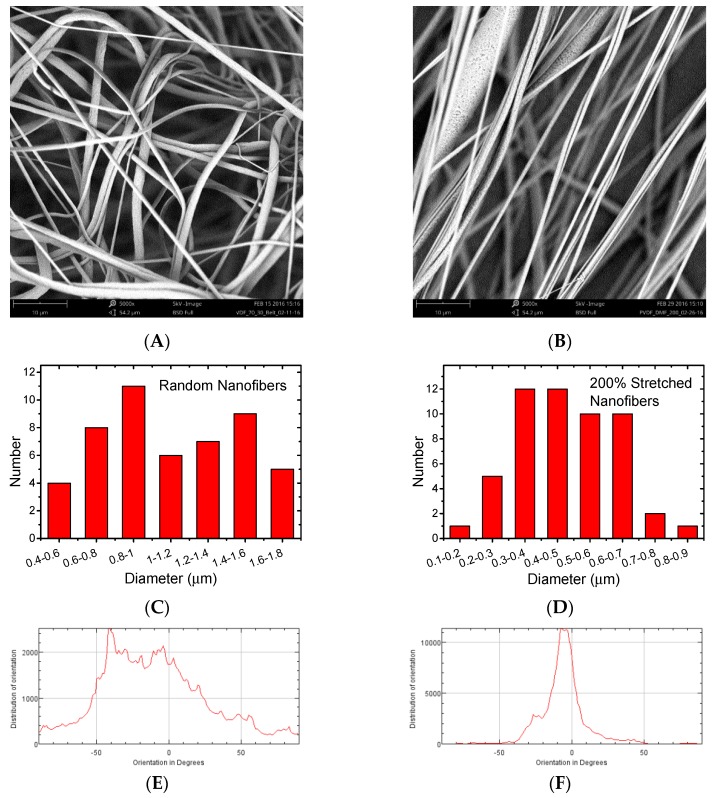
SEM images of (**A**) traditionally prepared electrospun PVDF-HFP nanofibers and (**B**) stretched PVDF-HFP nanofibers. Diameter distribution histograms of (**C**) random nanofibers and (**D**) stretched nanofibers. Nanofiber orientation distribution of (**E**) random nanofibers and (**F**) stretched nanofibers.

The diameter and alignment characterization results of the PVDF-HFP nanofibers are illustrated in Figure 7. In particular, Figure 7A shows an SEM image of a PVDF-HFP nanofiber sample prepared with the traditional electrospinning method. The nanofibers form a random network with no obvious orientation. Figure 7B shows an SEM image of the stretched electrospun nanofibers. The alignment of the stretched nanofibers is significantly enhanced. The diameters and their distribution were examined for both samples using the ImageJ software (Version: 1.50i, Wayne Rasband, U.S. National Institute of Health, Bethesda, MA, USA). Figure 7C,D show the nanofiber diameter distribution histograms of the two samples. For the electrospun PVDF-HFP nanofibers, a majority of the diameters are between 600 and 1600 nm; while the stretched fibers have their diameters clustered between 300 and 700 nm. The results demonstrate that the stretched nanofibers have smaller fiber diameters. Moreover, their fiber diameters are confined in a narrower range. Figure 7E,F display the nanofiber orientation distribution charts for the two samples based on the analysis results from ImageJ. The random nanofibers cover a wider range in terms of orientation in degrees, showing multiples peaks between −50° and 50°. The stretched nanofibers have their orientation agglomerated around 0° with almost no data points beyond the range of −50° to 50°. This shows that the stretching process can result in better aligned nanofibers.

### 3.3. Energy Harvesting Measurement

**Figure 8:**
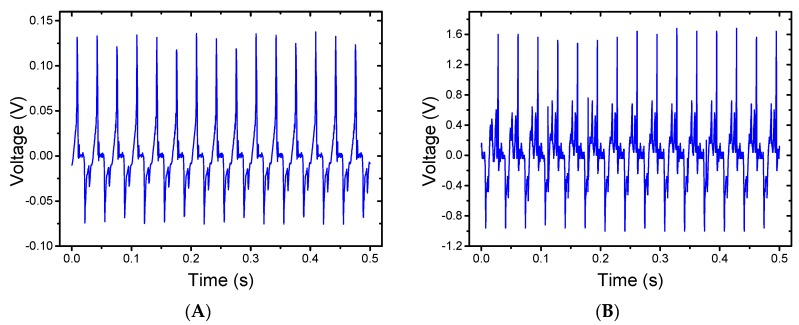
(**A**) Electrical output of a PDMS/PVDF-HFP/PDMS energy harvester using random nanofibers. (**B**) Electrical output of a device using aligned nanofibers.

Both random and stretched PVDF-HFP nanofiber samples were tested under the same conditions. Figure 8A shows the voltage generated from a PDMS/PVDF-HFP/PDMS device containing random nanofibers. The output signal shows a peak-to-peak voltage of approximately 0.21 V. Figure 8B shows the voltage generated form a device containing 200% stretched nanofibers. The output of the device shows a much higher peak-to-peak voltage of 2.6 V. This is a significant increase in terms of electrical output energy. The stretched nanofibers generate more than 12 times the voltage than that of the electrospun random nanofibers.

This voltage increase can be attributed to two factors. The enhanced fiber alignment increases the overall piezoelectricity of the sample. For a random nanofiber network, the dipoles are oriented in many different directions, as illustrated in Figure 7E. The electrical output signals induced by the mechanical stress have the same orientation distribution. The longitudinal piezoelectric effect along the force acting vector contributes to the output signals while the transverse piezoelectric effect perpendicular to that direction has little effect on the electrical output. The overall piezoelectric effect is, therefore, reduced by these random directions. In contrast, the aligned nanofibers have most of their electrical dipoles arranged along the same direction. Most of these nanofibers produce longitudinal piezoelectric effects under stress. This results in an accumulative effect and the overall piezoelectricity of the sample is, therefore, enhanced.

One key advantage of using electrospinning over other casting methods in preparing PVDF or its copolymers is that the strong electrical field used in the process provides in-situ alignment of the PVDF molecular structure. A combined effect from both mechanical stretching and electrical poling becomes a part of the preparation process [35,36,37]. This results in self-aligned electrical dipoles which in turn provide the material high piezoelectricity. Unlike many other piezoelectric materials, electrospun PVDF nanofibers do not require any further electrical poling steps. The stretching process used in our research provides an additional poling effect from the mechanical elongation of the nanofibers.

**Figure 9:**
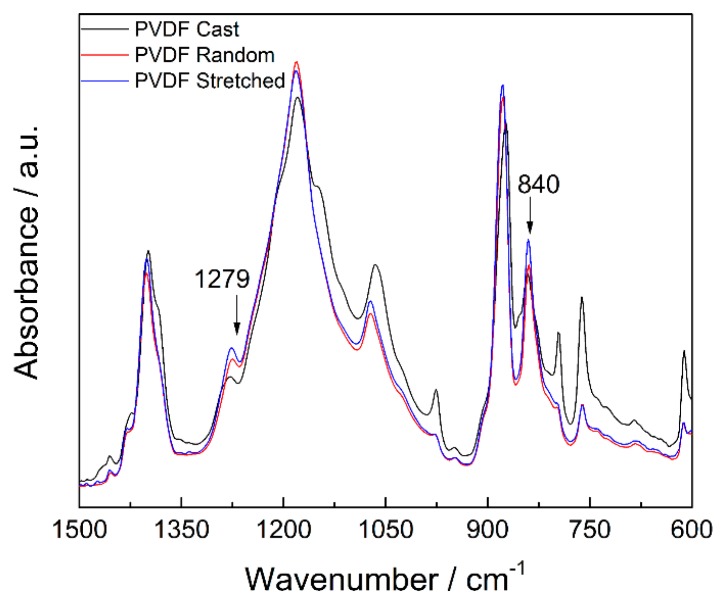
Normalized FTIR spectra of a cast PVDF-HFP film, random nanofibers, and aligned nanofibers.

The reduction in diameter indicates that the nanofibers have undergone stretching, which can induce molecular restructuring of the material. The FTIR spectra of the PVDF-HFP samples can provide valuable information about their structures and allow close examination of different crystalline forms. Figure 9 shows the FTIR spectra of the random and stretched nanofibers as well as a cast PVDF-HFP film as a control substrate. The cast film (directly casted and dried from a PVDF-HFP solvent) shows a very small β phase peak at 1279 cm^−1^. By comparison, both electrospun PVDF nanofiber samples show clear β phase peaks. The stretched nanofibers have a higher peak than the random nanofibers. This proves that the stretching process can enhance the β phase content in electrospun nanofibers. We anticipate that further stretching beyond 200% can potentially increase the β phase content in the nanofibers. Further material characterization is currently underway to examine nanofibers with various stretching ratios with known increments. It is expected that the new results will provide more quantitative information on the crystal structures and molecular properties of the electrospun nanofibers.

## 4. Conclusions

In conclusion, we have developed a novel energy harvester by integrating electrospun PVDF-HFP copolymer nanofibers with flexible substrates. Random nanofibers are prepared using a traditional electrospinning process and stretched/aligned nanofibers are prepared with an additional step of controlled stretching. The electro-mechanical testing demonstrates that the stretched PVDF-HFP nanofibers demonstrate higher piezoelectricity and are, therefore, more suitable for energy harvesting applications. The SEM inspection shows that the stretched nanofibers are better aligned with more precise diameter control. The mechanical-to-electrical conversion of the stretched nanofibers outperform the random sample by more than 10 times. The FTIR characterization reveals that the β phase content in the stretched nanofibers is enhanced. Because the β phase is considered the most critical polymorph that enables piezoelectricity in PVDF, we believe that our results demonstrate a promising approach for the fabrication of high-performance energy harvesters. In addition, we investigated the properties of various materials that can potentially be used as the flexible substrate in wearable systems. As PDMS is initially used in the assembled device with satisfactory performance, a new SF-glycerol composite material was investigated as an alternative with better biocompatibility and a more suitable candidate for medical-related areas. The characterization results show that the SF-glycerol composite can provide higher strength and larger ultimate strain than PDMS. The combination of high-performance piezoelectric polymers, enhanced energy harvesting capability, biocompatible silk composites, and device flexibility is anticipated to open new opportunities for wearable electronics research.

## Supplementary Materials

The following are available online at www.mdpi.com/2073-4360/9/10/479/s1, Figure S1: Standard DSC scans of Mori SF film and Mori SF-glycerol composite film. The samples were heated at 2 °C/min from −25 °C to 400 °C with temperature regions related to bound water evaporations (*T*_w_) and sample degradation (*T*_d_). Figure S2: Reversing heat capacities of the Mori SF film and Mori SF-glycerol composite film obtained from TMDSC with 2 °C/min heating rate, a modulation period of 60 s, and temperature amplitude of 0.318 K. Table S1: Comparison of the mechanical properties of PDMS, SF and SF-glycerol.
