# Supplementary material for: Biocompatible Silk/Polymer Energy Harvesters Using Stretched Poly (vinylidene fluoride-co-hexafluoropropylene) (PVDF-HFP) Nanofibers
Source: Polymers (Basel). 2017 Sep 30;9(10):479. doi: 10.3390/polym9100479 (PMC6418575; doi:10.3390/polym9100479)
Supplement: Supplementary file 1 [file polymers-09-00479-s001.pdf]

Supplementary Materials: Biocompatible Silk/Polymer Energy Harvesters Using Stretched Poly (vinylidene fluoride-co-hexafluoropropylene) (PVDF-HFP) Nanofibers

Raghid Najjar, Yi Luo, Dave Jao, David Brennan, Ye Xue, Vince Beachley, Xiao Hu and Wei Xue

The thermal properties of the SF film and SF-glycerol film samples were also examined by standard DSC and temperature modulated DSC (TMDSC). Figure S1 shows the standard DSC scans of the Mori SF film and Mori SF-glycerol composite film. The Mori silk film showed a small bound water peak around 97 °C, and Mori SF-glycerol composite showed a lower bound water peak around 70 °C. After the bound water evaporated, both samples showed degradation peaks, 259 °C for Mori silk film and 198 °C for Mori SF-glycerol composite film, indicating that both samples are thermally stable in the regular temperature range for our sensor applications. Temperature modulated DSC was used to measure the reversing thermal properties of Mori SF film and Mori SF-glycerol film.

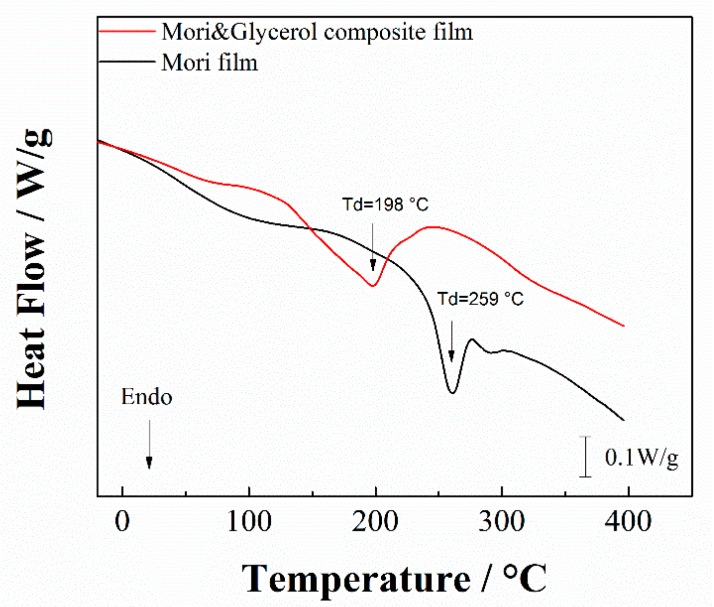

Figure S1. Standard differential scanning calorimetry (DSC) scans of Mori SF film and Mori SF-glycerol composite film. The samples were heated at 2 °C/min from −25 °C to 400 °C with temperature regions related to bound water evaporations (T_w) and sample degradation (T_d).

Figure S2 shows the reversing heat capacity of both samples. The glass transition region usually appears as a step in the baseline of the recorded DSC curve. A clear glass transition appeared for both samples, 173 °C for Mori SF film and 135 °C for Mori SF-glycerol composite film, indicating is no significant physical property change for the sensor substrate (either SF or SF-glycerol) when the temperature is below 135 °C. The homogeneous glass transition regions for the Mori SF-glycerol composite film also indicated there is no micro-phase separate in the composite film when glycerol content is 20 wt%.

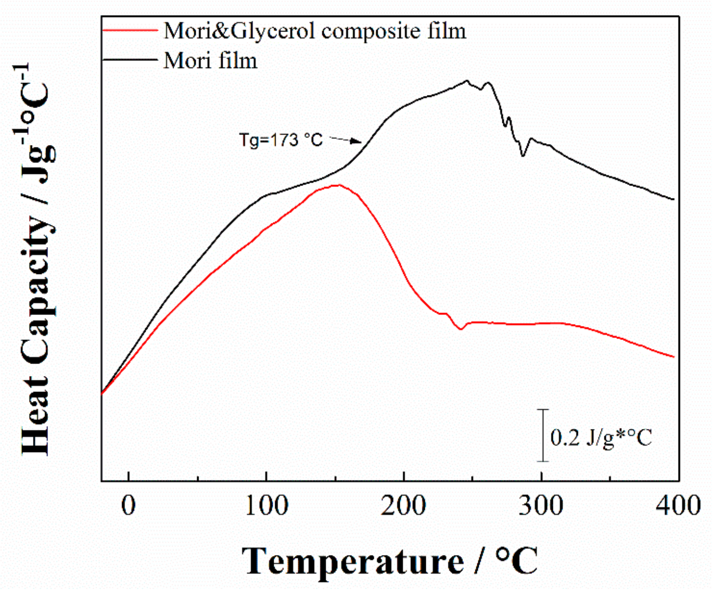

Figure S2. Reversing heat capacities of the Mori SF film and Mori SF-glycerol composite film obtained from temperature modulated differential scanning calorimetry (TMDSC) with 2 °C/min heating rate, a modulation period of 60 s, and temperature amplitude of 0.318 K.

Table S1 summarizes the mechanical properties of PDMS, pure SF, and SF-glycerol samples. The values are obtained from the stress-strain curves of these three materials.

Table S1. Comparison of the mechanical properties of PDMS, SF, and SF-glycerol.

Mechanical Properties	PDMS	Silk Fibroin	Silk Fibroin + 20% Glycerol
Young's Modulus (MPa)	0.1062	4.7892	1.1621
Yield Strength (MPa)	0.4640	2.0833	1.3684
Yield Strain	4.3675	0.4350	1.1775
Ultimate Strength (MPa)	0.4640	6.2500	2.6711
Ultimate Strain	4.3675	9.5450	13.6375
